# EnZolv delignification of cotton spinning mill waste and optimization of process parameters using response surface methodology (RSM)

**DOI:** 10.1186/s13068-024-02473-w

**Published:** 2024-03-07

**Authors:** Santhoshkumar Subramaniam, Kumutha Karunanandham, A. S. M. Raja, S. K. Shukla, Sivakumar Uthandi

**Affiliations:** 1 Biocatalysts Laboratory, Department of Agricultural Microbiology, Tamil Nadu Agricultural University (TNAU), Coimbatore, Tamil Nadu 641003 India; 2 Department of Agricultural Microbiology, Agricultural College and Research Institute, Madurai, Tamil Nadu 625104 India; 3 ICAR-Central Institute for Research on Cotton Technology, Adenwala Road, Matunga, Mumbai, 400019 India

**Keywords:** Cotton spinning mill waste, EnZolv, Laccase, Optimization, Pretreatment, Response surface methodology

## Abstract

**Background:**

EnZolv is a novel enzyme-based, eco-friendly biomass pretreatment process that has shown great potential in the field of textile engineering and biotechnology. It employs laccase from *Hexagonia hirta* MSF2 and 2% ethanol in the process of delignification. The process is designed to evaluate optimal conditions to remove lignin and other impurities from cotton spinning mill waste (CSMW), without compromising the quality and strength of the fibers. CSMW is a low-cost and readily available source of cellulose, making it an ideal candidate for delignification using EnZolv. By optimizing the pretreatment conditions and harnessing the potential of enzymatic delignification, this research aims to contribute to more sustainable and efficient ways of utilizing lignocellulosic biomass in various industries for the production of biochemical and bioproducts.

**Results:**

The present study emphasizes the EnZolv pretreatment in the delignification of cotton spinning mill wastes irrespective of the cellulose content. EnZolv process parameters such as, moisture content, enzyme load, incubation time, incubation temperature, and shaking speed were optimized. Under pre-optimized conditions, the percent lignin reduction was 61.34%, 61.64%, 41.85%, 35.34%, and 35.83% in blowroom droppings (BD), flat strips (FS), lickerin fly (LF), microdust (MD) and comber noils (CN), respectively. Using response surface methodology (RSM), the statistically optimized EnZolv pretreatment conditions showed lignin reduction of 59.16%, 62.88%, 48.26%, 34.64%, and 45.99% in BD, FS, LF, MD, and CN, respectively.

**Conclusion:**

Traditional chemical-based pretreatment methods often involve harsh chemicals and high energy consumption, which can have detrimental effects on the environment. In contrast, EnZolv offers a greener approach by utilizing enzymes that are biodegradable and more environmentally friendly. The resulting fibers from EnZolv treatment exhibit improved properties that make them suitable for various applications. Some of the key properties include enhanced cellulose recovery, reduced lignin content, and improved biophysical and structural characteristics. These improvements can contribute to the fiber's performance and processability in different industries and future thrust for the production of cellulose-derived and lignin-derived bioproducts.

**Graphical Abstract:**

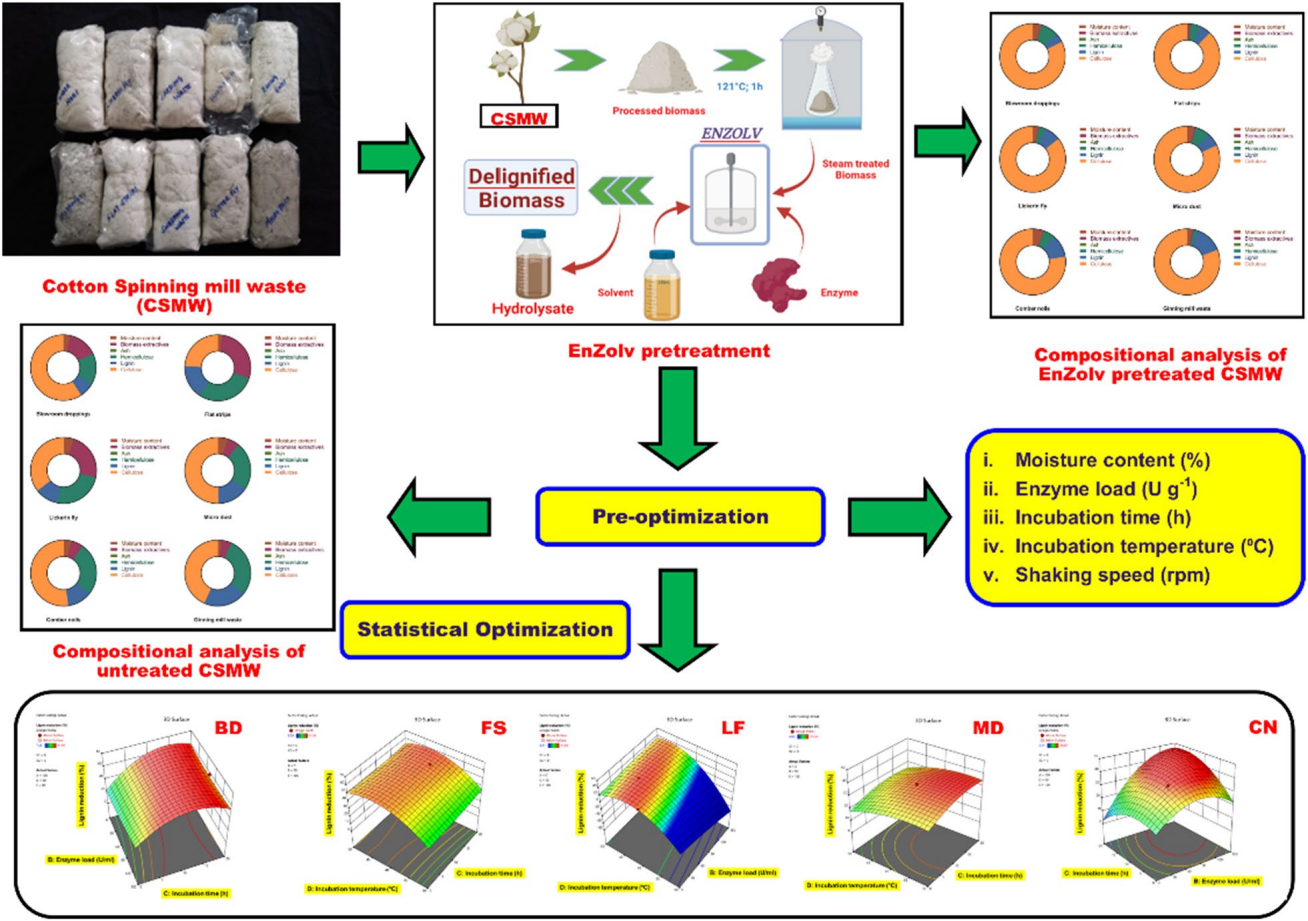

**Supplementary Information:**

The online version contains supplementary material available at 10.1186/s13068-024-02473-w.

## Background

Cotton, the most widely available natural fiber, meets the demand of the global textile industry. Every year, the world produces more than 27.5 million metric tonnes (MMT) of cotton, with 27.0 MMT specifically used for textile production, making the cotton sector a significant contributor to the global economy. The top cotton-producing nations, including India (5.53 MMT), China (4.99 MMT), the United States of America (3.72 MMT), Brazil (1.72 MMT), and Pakistan (1.54 MMT), account for 75% of global cotton production [1]. In 2018, cotton held a market share of approximately 39.47% in the textile fiber industry [2]. It is projected that India will produce 311.18 lakh bales (1 bale = 170 kg) of cotton during the 2022–2023 growing season [3]. Growing environmental concerns related to global warming have led to an increasing interest in bioenergy as a renewable energy source, primarily due to the extensive use of fossil fuels [4]. The sustainable development goals (SDGs) framework provides a standardized method for devising strategies that address specific issues related to energy, and environment [5]. The bioeconomy concept emerges from the necessity to mitigate the harm caused by non-renewable resources. This concept also aligns with the SDGs since a transition towards a bioeconomy reduces the dependence on non-renewable resources [6]. The Food and Agriculture Organization of the United Nations (FAO) defines the bioeconomy as the production of renewable biological resources and their conversion into value-added products, such as food, feed, bio-based products, and bioenergy. Biorefineries are potential solutions for developing a circular bioeconomy that utilize biomass efficiently and support the SDG’s concept. Biomass-based biofuels have minimal impact on the greenhouse effect due to their renewable nature. Residual biomass, in particular, is poised to become an important alternative energy source with carbon-neutral properties [7]. However, a significant challenge lies in efficiently handling, converting, and storing agricultural waste into bioenergy products due to their structural variability, irregular physical properties, low energy density, high moisture content, and hydrophilic nature. Additionally, the direct burning of agricultural waste in residential and commercial settings leads to inefficiency and severe air pollution [8].

Lignocellulosic biomass (LCB) is a renewable and abundant source of organic matter that can be converted into various value-added products such as biofuels, chemicals, and materials. However, LCB has a complex and recalcitrant structure that hinders its efficient utilization. Therefore, pretreatment of LCB is a crucial step to overcome its physical and chemical barriers, and to enhance its accessibility and digestibility for subsequent processes. Pretreatment of LCB can be classified into four main categories: physical, chemical, physicochemical, and biological. Physical pretreatment methods are generally simple and fast, but they require high energy input and may not be sufficient to disrupt the lignin barrier [9]. Chemical pretreatment methods are usually effective and versatile, but they generate toxic by-products detrimental to the environment upon release and require high capital and operating costs [10]. The formation of inhibitory compounds and added cost for solvent removal are serious drawbacks of other methods like organosolv and deep eutectic solvents [11]. Biological pretreatment methods employ microorganisms or enzymes to degrade or modify some components of LCB, mainly lignin, and hemicellulose, and enhance the enzymatic hydrolysis of cellulose. This method of pretreatment is environmentally friendly and specific, but they are slow and requires strict control of operating conditions [9]. EnZolv, a greener pretreatment approach facilitates the removal of lignin from LCB with steam and laccase (LccH) in the presence of an organic solvent that generates valuable chemicals in the hydrolysate [12]. Moreover, many studies have applied laccase and mediator systems for enhanced lignin removal and optimized the process conditions for higher yield. Unlike other green pretreatment methods, EnZolv offers the benefit of simultaneous delignification and valuable chemical production in a single process. Furthermore, EnZolv pretreatment improves the performance of LccH under solvents that serve as an inducer to enhance laccase activity for higher lignin removal and concurrent production of valuable chemicals to fulfill the lignin-first biorefinery concept. Solvent (2% ethanol) used in EnZolv delignification provides stability to the laccase enzyme since the EnZolv is a time-dependent process. Laccases represent the most adaptable oxidoreductases, relying on oxygen for their functionality. The increased solubility of oxygen in organic solvents compared to water facilitates easier access to electrons through the oxidation of the reduced substrate [12].

Lignin is a complex molecule with a cross-linking structure and contains various functional groups, including aliphatic hydroxyl, phenolic hydroxyl, and methoxyl groups. These functional groups, especially hydroxyl groups and the aromatic structure, play important roles in determining the characteristics of lignin and its reactivity. The three main precursors of the lignin polymer are coniferyl alcohol, sinapyl alcohol, and *p*-coumaryl alcohol. Different plants utilize these precursors in different proportions, resulting in variations in the composition of lignin [13]. There are two main types of linkages in lignin: carbon–carbon (C–C) linkages and ether linkages. Ether linkages, particularly β-O-4′ ether bonds, are the most prevalent type, accounting for the majority of linkages in lignin [14, 15]. Laccase is an oxidative enzyme that can break down the ether linkages in lignin. It is considered to be environmentally friendly and efficient in lignin degradation. Laccase selectively targets the phenolic component of lignin through oxidation reactions. Compared to other enzymes, laccase has high catalytic activity and the ability to carry out non-specific oxidations. It does not require the use of hydrogen peroxide and converts molecular oxygen to water. Laccase has shown potential for use in LCB pretreatment due to its substrate specificity [16]. Previous studies using a laccase-based hydrothermal cavitation reactor for corn cob pretreatment achieved a significant lignin removal [17].

To produce textile yarns, cotton is put through several processes, including ginning, spinning, warping, slashing, knitting, weaving, preparation, de-sizing, bleaching, dyeing, printing, and finishing [18]. Blowroom and carding area generate about 8% of waste, while the combing section can generate up to 20% of waste [19, 20]. In rotor spinning, these wastes are employed to create coarser yarns for denim and jeans. Additionally, several equipments used in the spinning line produce more than 1% of the cotton fly during processing [21].

The presence of impurities in cotton, such as lignin-containing elements like seeds and cotton shells, is a common occurrence despite cotton being a rich source of cellulose. These impurities primarily stem from issues during the picking of cotton bolls, separation, and ginning processes. The objective of current efforts is to make use of the waste or unusable products generated at different stages of the spinning mill processes. These waste products are treated as a biomass source for EnZolv pretreatment to remove lignin, which is a recalcitrant and integral component of biomass. Cotton waste, being mainly composed of cellulose, is the focus of this study to achieve the concept of a circular economy. The ultimate goal is to remove lignin from the waste biomass, enabling its utilization in various applications such as the production of cellulose, derived chemicals, and biofuels.

## Materials

### Sample collection

Cotton spinning mill wastes (CSMW) were collected from Veejay Syntax Pvt. Ltd, Kottaipalayam, Coimbatore, India. The waste includes blowroom droppings (BD), flat strips (FS), lickerin fly (LF), microdust (MD), and comber noils (CN). All the wastes were collected afresh during the spinning process and transported to the Biocatalysts Laboratory, Department of Agricultural Microbiology, Tamil Nadu Agricultural University, Coimbatore, for further studies.

### Pre-optimization of EnZolv pretreatment

The EnZolv method was applied to cotton spinning mill wastes (CSMW) as a pretreatment technique that combines steam and biocatalysis [12]. The CSMW was exposed to steam at 121 °C and 15 psi for 1 h, and mixed with a solvent (2% ethanol), a buffer (citrate phosphate, pH 3.4), and crude laccase enzyme (LccH) (50 U g^−1^ of dry biomass) from *Hexagonia hirta* MSF2. The mixture was incubated at 40 °C and 120 rpm for 17 h. The filtered biomass was then analyzed for its composition using the NREL standards [22]. Based on the previous study that employed EnZolv pretreatment of *Melia dubia*, a woody biomass [12], the process parameters of EnZolv pretreatment of CSMW were pre-optimized for its moisture content (0–100%), enzyme load (0–150 U), incubation time (0–20 h), incubation temperature (30–50 °C), and shaking speed (0–180 rpm). These process parameters were chosen for pre-optimization and the parameter with maximum delignification will be selected for further bioconversion and product recovery.

### Optimization of EnZolv parameters by response surface methodology

RSM is a technique that uses mathematical and statistical methods to study how the output response is influenced by the interactions of the process variables. RSM has the advantages for optimizing the process with fewer experiments, saving time, materials, and resources, and also allowing for the interpretation of the relationship between the independent variables [23]. Moreover, RSM can reveal the interactions between the variables, and show how they affect the response. This study shows the experimental design that was used to evaluate the effect of five different process factors on the lignin reduction percentage, namely moisture content (A), enzyme load (B), incubation time (C), incubation temperature (D), and shaking speed (E). The Box–Behnken design option of the Design Expert^®^ 10.0 software (Stat-Ease, Inc.) was used to generate the different experimental scenarios.

The removal of lignin from raw biomass is studied as a part of optimizing the biomass pretreatment. The EnZolv pretreatment involves many factors that affect the lignin breakdown, such as moisture content, enzyme load, incubation time, temperature, and shaking rate. Each of these five independent factors has an influence on the result (i.e., % lignin reduction). The ranges of these independent variables were fixed based on the pre-optimization experiments, as follows: (A) moisture content (0–100%), (B) enzyme load (0–150 U g^−1^ of biomass), (C) incubation time (0–20 h), (D) incubation temperature (30–50 °C), and (E) shaking speed (0–180 rpm). These values were chosen based on preliminary tests that showed satisfactory product yields. The Design-Expert software^®^ 10.0 (Stat-Ease, Inc., USA) was used to generate 46 experimental runs with five different variables using the Box–Behnken design (BBD) [24].

## Results

### Proximate and compositional analysis of CSMW

**Fig. 1:**
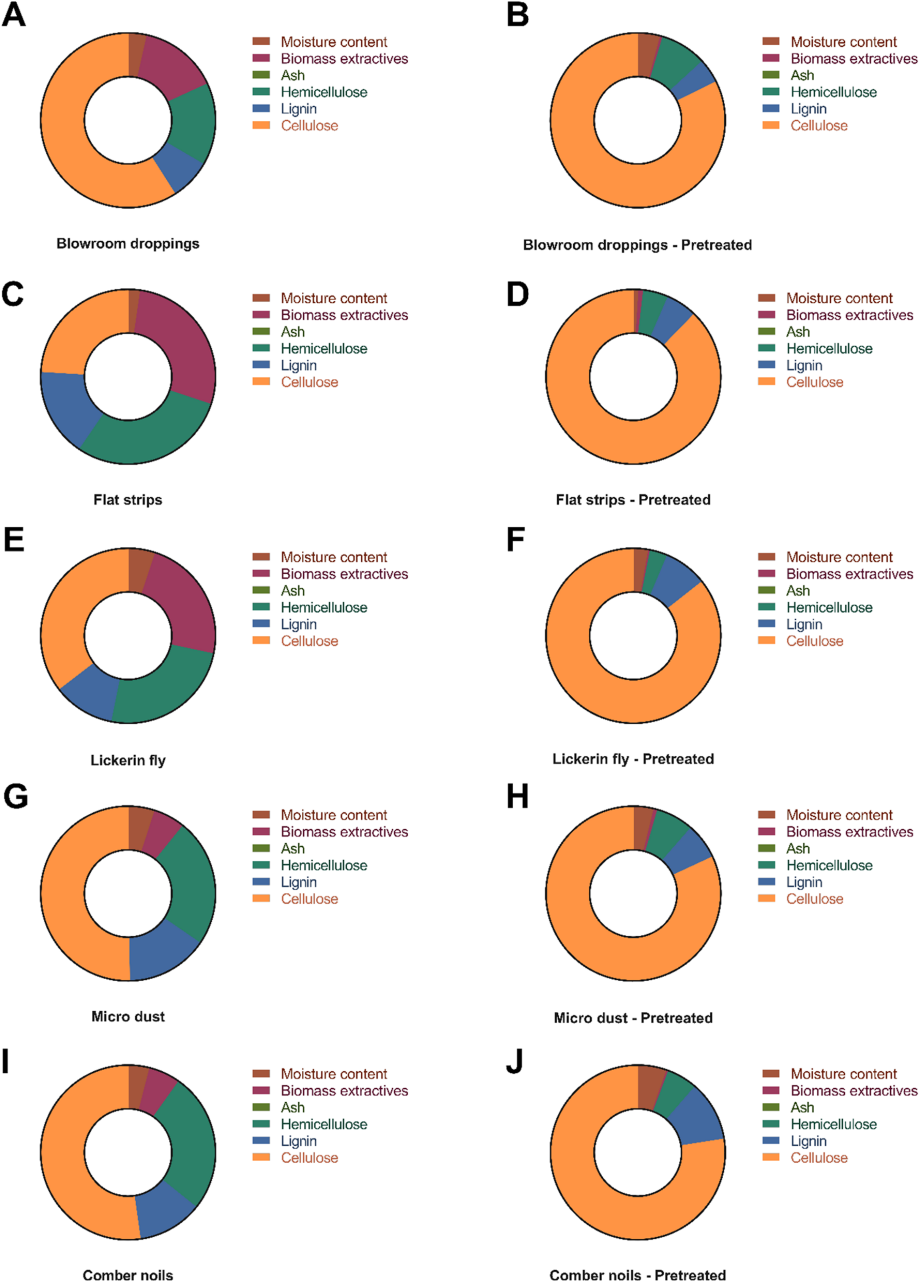
Donut chart indicating the compositional parts of the whole CSMW biomass. **A** Untreated blowroom droppings; **B** conventionally EnZolv pretreated blowroom droppings; **C** untreated flat strips; **D** conventionally EnZolv pretreated flat strips; **E** untreated lickerin fly; **F** conventionally EnZolv pretreated lickerin fly; **G** untreated microdust; **H** conventionally EnZolv pretreated microdust; **I** untreated comber noils; **J** conventionally EnZolv pretreated comber noils

Agroresidue-based lignocellulosic biomass has great market potential because of its surplus availability and its structural integrity is protected by lignin, cellulose, and hemicellulose as their primary constituents. Initial compositional analyses of untreated CSMW biomass revealed the lignin content of BD, FS, LF, MD, and CN was 7.56%, 16.40%, 11.40%, 15.15%, and 11.99%, hemicellulose content of 15.27%, 29.43%, 24.83%, 23.77%, and 25.93%, and cellulose content of 59.04%, 24.05%, 35.43%, 50.27%, and 52.24%, respectively (Fig. 1).

### EnZolv pretreatment of CSMW

Pretreatment is the most crucial stage in the delignification process since lignin is naturally recalcitrant. EnZolv is regarded as one such pretreatment technique since it is a more eco-friendly and greener method of pretreatment. In the present study, the pretreated BD biomass has 2.92% lignin, 88.45% cellulose, and 2.60% hemicellulose. Similarly, pretreated FS with EnZolv demonstrates 6.29% lignin, 88.32% cellulose, and 4.10% hemicellulose. EnZolv pretreated LF, comprised 6.63% lignin, 91.31% cellulose, and 1.60% hemicellulose. Pretreated MD had 9.79% lignin, 84.76% cellulose, and 3.60% hemicellulose. In its pretreated biomass, CN has 7.69% lignin, 87.32% cellulose, and 1.35% hemicellulose (Fig. 1). The lignin content from untreated and conventional EnZolv pretreated CSMW showed 61.38%, 61.65%, 41.84%, 35.38%, and 35.86% lignin removal from BD, FS, LF, MD, and CN, respectively. Similarly, cellulose content increased to 33.24%, 72.77%, 61.20%, 40.69%, and 40.17% in BD, FS, LF, MD, and CN samples due to EnZolv pretreatment. The diverse composition observed before and after pretreatment stems from the modification of cotton biomass at various stages in the spinning process. These steps induce changes in the size, texture, and properties of fibers, thus significantly impacting the EnZolv pretreatment.

### Pre-optimization of EnZolv process in CSMW

**Fig. 2:**
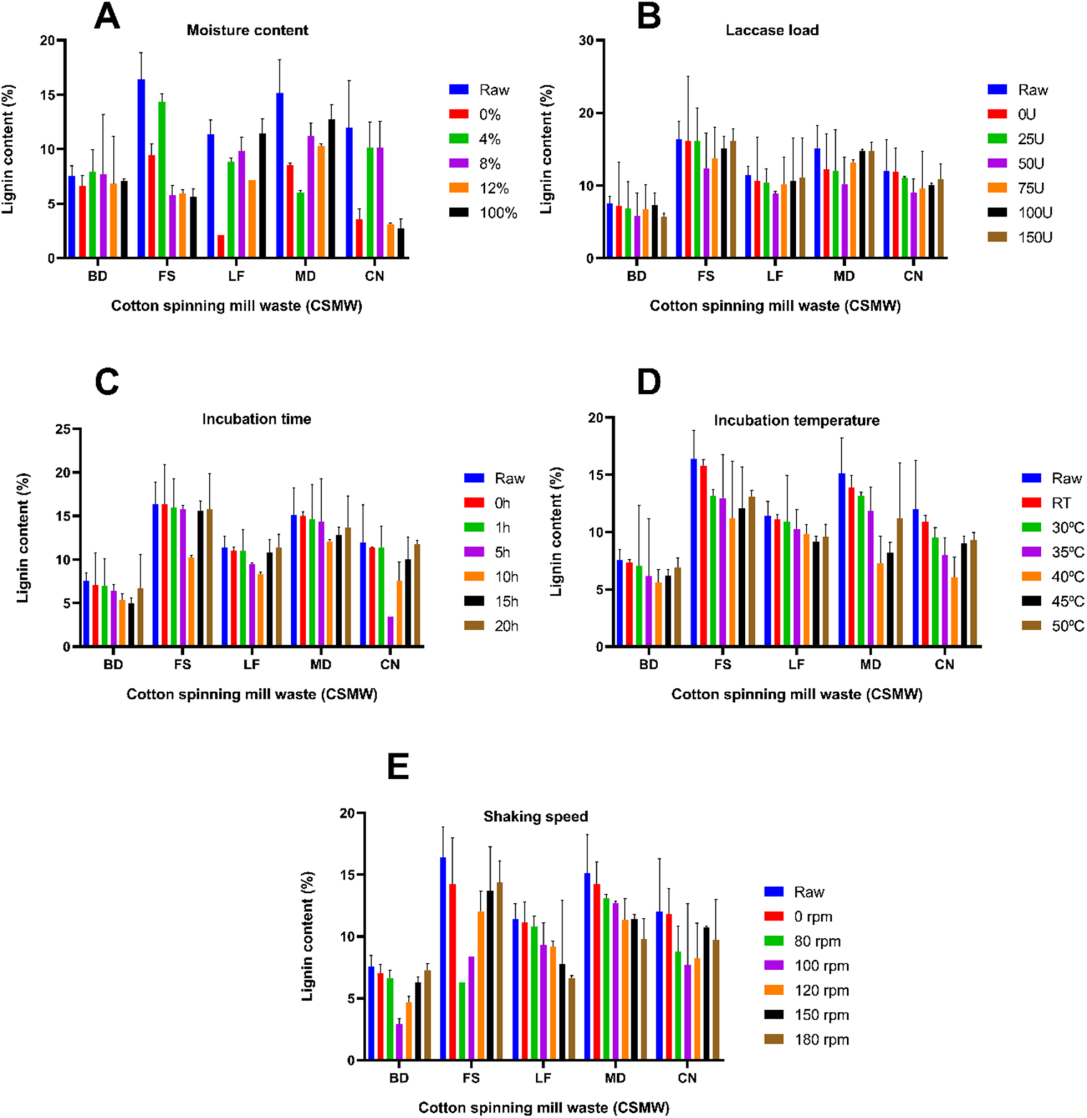
Lignin content (%) of the CSMW under EnZolv pre-optimization conditions. **A** Moisture content; **B** enzyme load; **C** incubation time; **D** incubation temperature; **E** shaking speed

Lignin reduction by the conventional EnZolv pretreatment was not substantial when the process was experimented in CSMW since the EnZolv process was first developed for *Melia dubia* woody biomass [12]. As a result, the process parameters were adjusted to maximize the lignin reduction potential of EnZolv pretreatment process in CSMW. Moisture content during pretreatment not only makes the *e* biomass more prone during steam treatment, but also makes enzyme accessible to lignin hydrolysis. Contrary to the aforementioned assertion, at 0% moisture content, the highest lignin reduction for CSMW was 11.81% and 80.70% for the samples BD and LF, respectively. At 100% moisture level, FS, and CN noted a lignin reduction of 65.22% and 76.72%, respectively. Lignin reduction of 60.30% was recorded for MD at 4% moisture content (Fig. 2a).

Enzyme load is another significant component that impacts the delignification of lignocellulosic biomass. Since laccase, an oxidative enzyme, acts on the lignin portion of the biomass and hydrolyzes it into lignin monomers and high-value generated aromatics, it is used in the EnZolv process. The findings of the enzyme load optimization show that the highest lignin reduction for all CSMW was obtained at 50 U g^−1^ of biomass with 22.52%, 24.35%, 21.27%, 32.40%, and 24.78% for the samples BD, FS, LF, MD, and CN, respectively (Fig. 2b). At different incubation times, EnZolv pretreatment of CSMW revealed that at 15 h, the highest reduction in lignin content in BD was 33.39%. Likewise, at 10 h incubation time, maximum lignin reduction in FS, LF, and MD was noted as 37.37%, 26.86%, and 20.11%, respectively. Comber noils exhibited a maximum lignin reduction of 71.12% at 5 h incubation time (Fig. 2c). Similarly, for optimization of incubation temperature in the EnZolv process, the samples BD, FS, MD, and CN exhibited maximum lignin reduction of 25.79%, 31.59%, 51.92%, and 49.32%, respectively at 40 °C. Lignin reduction of 19.38% was recorded at 45 °C for lickerin fly (Fig. 2d).

**Fig. 3:**
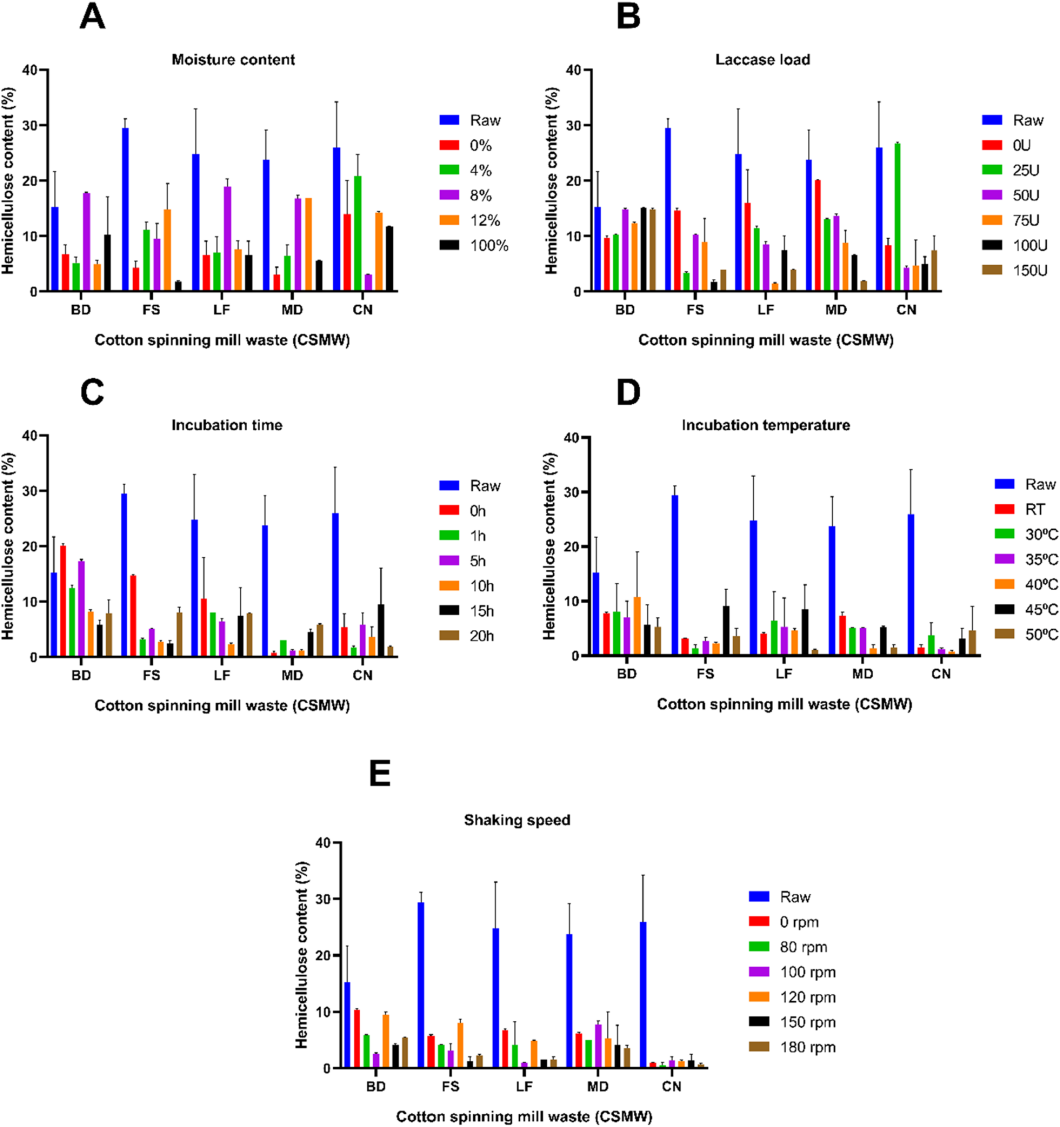
Hemicellulose content (%) of the CSMW under EnZolv pre-optimization conditions. **A **Moisture content; **B** enzyme load; **C** incubation time; **D** incubation temperature; **E** shaking speed

The EnZolv process for CSMW at various shaking speeds showed that BD and CN had a maximum lignin reduction of 61.34% and 35.86%, respectively, at 100 rpm. Similarly, at 80 rpm, FS had its lignin level reduced by 61.64%. At 180 rpm, LF, and MD exhibited lignin reduction of 41.85% and 35.34%, respectively (Fig. 2e). Cellulose recovery and hemicellulose reduction for the optimized EnZolv pretreatment parameters were noted for CSMW. The hemicellulose content was determined to be 2.6%, 4.1%, 1.6%, 3.6%, and 1.35% for BD, FS, LF, MD, and CN, respectively, based on the pre-optimized conditions. The EnZolv-optimized CSMW samples such as BD, FS, LF, MD, and CN showed hemicellulose reductions of 82.97%, 28.07%, 93.56%, 84.85%, and 94.79%, respectively (Fig. 3). EnZolv pretreatment has been proved to be a more effective and environmentally friendly procedure when compared to the physical and acid pretreatment methods.

**Fig. 4:**
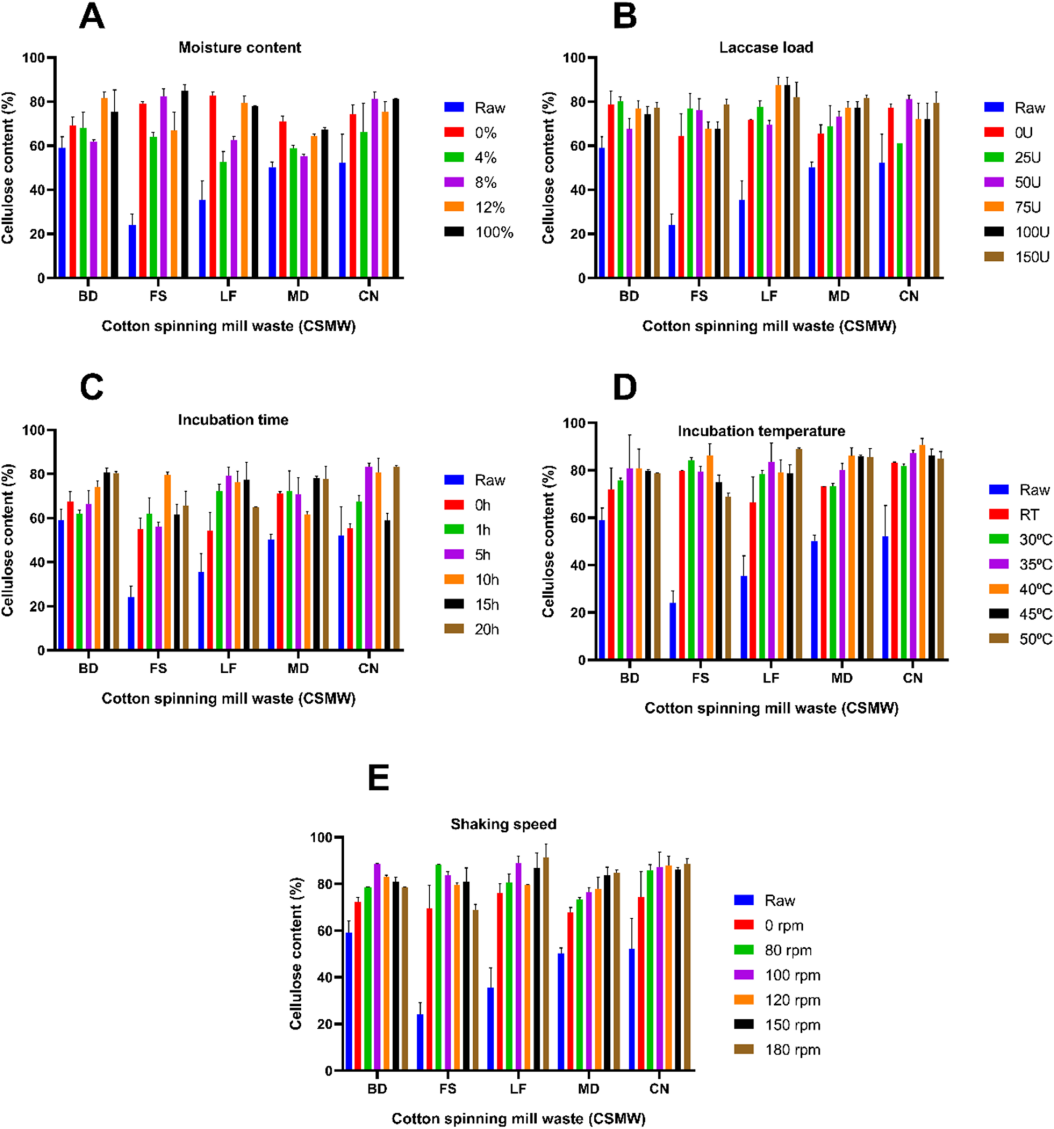
Cellulose content (%) of the CSMW under EnZolv pre-optimization conditions. **A** Moisture content; **B** enzyme load; **C** incubation time; **D** incubation temperature; **E** shaking speed

The cellulose chains are frequently organized into microfibrils, which are tightly packed bundles of cellulose encased in a coating of hemicelluloses and lignin, to block access to the chains [25]. After EnZolv pretreatment, cellulose recovery from CSMW was determined under pre-optimized conditions. According to the findings, the cellulose content increased in BD, FS, LF, MD, and CN from 59.04–88.45%, 24.05–88.32%, 35.43–91.31%, 50.27–84.76%, and 52.24–87.32%, registering a percentage increase in cellulose content of 33.25%, 72.77%, 61.20%, 40.69%, and 40.17%, respectively (Fig. 4).

### Statistical optimization of CSMW using response surface methodology

EnZolv pretreatment conditions of the cotton spinning mill waste (CSMW) were optimized using the Box–Behnken design for varied moisture content, enzyme load, incubation time, incubation temperature, and shaking speed and lignin reduction (%) as their response. The second-order equation in terms of coded factors generated by Box–Behnken design for EnZolv pretreatment optimization process in blowroom droppings (BD) is

$${\text{Lignin reduction }}\, \left( \% \right) = 52.21 - 2.11A + 0.7375B + 1.65C + 7.06D + 2.27E + 0.1994AB - 0.4257AC + 4.81AD - 0.1294AE + 1.03BC - 22.81BD - 0.0248BE - 0.0877CD + 1.03CE - 6.55DE + 1.06A^{2} - 2.06B^{2} + 1.33C^{2} - 13.34D^{2} - 1.74E^{2} .$$

Similarly, the second-order equation in terms of coded factors generated by Box–Behnken design for EnZolv pretreatment optimization process in flat strips (FS) is

$${\text{Lignin}}\, {\text{reduction}}\, \left( \% \right) = 52.90 + 4.34A + 2.25B - 2.39C - 0.8126D - 4.20E + 0.5465AB + 15.42AC - 3.31AD - 0.1103AE - 5.53BC + 9.17BD - 4.04BE - 6.31CD - 4.28CE + 3.39DE + 5.97A^{2} + 3.10B^{2} - 22.64C^{2} - 9.85D^{2} - 6.29E^{2} .$$

The second-order equation in terms of coded factors generated by Box–Behnken design for EnZolv pretreatment optimization process in lickerin fly (LF) is

$${\text{Lignin reduction}}\, \left( \% \right) = 30.49 + 1.27A - 2.41B + 8.53C + 30.80D + 1.74E + 3.94AB + 3.25AC - 0.3987AD + 0.1294AE - 10.98BC + 16.70BD - 3.33BE - 14.70CD - 6.01CE + 5.56DE + 1.01A^{2} - 3.35B^{2} - 4.80C^{2} - 38.05D^{2} + 0.3510E^{2} .$$

The second-order equation in terms of coded factors generated by Box–Behnken design for EnZolv pretreatment optimization process in microdust (MD) is

$${\text{Lignin reduction}}\, \left( \% \right) = 18.06 - 0.7856A + 3.15B + 1.42C + 0.6196D + 5.29E + 1.18AB - 3.03AC + 3.23AD + 0.1476AE + 2.24BC + 1.98BD - 2.69BE - 0.0285CD + 0.2870CE - 1.65DE + 2.95A^{2} - 2.32B^{2} - 4.12C^{2} - 3.82D^{2} + 7.70E^{2} .$$

The second-order equation in terms of coded factors generated by Box–Behnken design for EnZolv pretreatment optimization process in comber noils (CN) is

$${\text{Lignin reduction}}\, \left( \% \right) = 34.31 + 1.04A + 1.33B + 5.78C + 2.72D + 8.19E + 9.93AB - 3.67AC - 7.13AD + 1.35AE + 9.51BC + 7.84BD + 10.77BE + 1.67CD + 3.70CE + 5.16DE + 6.87A^{2} - 12.52B^{2} - 8.90C^{2} - 4.53D^{2} - 4.88E^{2} ,$$

where *A* is the moisture content in %, *B* is the enzyme load in U g^−1^ of biomass, *C* is the incubation time in hours, *D* is the incubation temperature in °C, and *E* is the shaking speed in rpm.

The ANOVA Table presents a quadratic model that fits the response data well and was produced by the EnZolv pretreatment conditions for blowroom droppings (Additional file 1: Table S1), flat strips (Additional file 1: Table S2), lickerin fly (Additional file 1: Table S3), microdust (Additional file 1: Table S4), and comber noils (Additional file 1: Table S5). The *F*-values were 19.21, 14.65, 16.10, 4.56, and 7.43 for BD, FS, LF, MD, and CN, respectively, and *p-*value (< 0.0001) except for MD (0.0002) of the model from the ANOVA test demonstrate the significance of the model variables presented and the low probability of noise (0.01%) associated with them. The incubation temperature in BD, LF, MD, and shaking speed in FS, CN has the greatest *F*-value among the five variable factors examined and is considered to be the influential variable for EnZolv pretreatment. The least significant variable is indicated by enzyme load in BD, incubation temperature in FS, moisture content in LF, and CN, and shaking speed in MD with the lowest *F*-value. According to the model *p-*values, the variables D, E, AD, BD, DE, and D^2^ in blowroom droppings (BD), A, E, AC, BD, CD, A^2^, C^2^, D^2^, and E^2^ in flat strips (FS), C, D, BC, BD, BE, CD, CE, B^2^, C^2^ in lickerin fly (LF), E, E^2^ in microdust (MD), C, E, AB, AD, BC, BE, A^2^, B^2^, C^2^ in comber noils (CN) are significant. The regression coefficient (*R*-square value) of the model, which was calculated to be 0.9389, 0.9214, 0.9279, 0.7847, 0.8560 is highly consistent with the adjusted *R*-square values of 0.8900, 0.8585, 0.8703, 0.6125, 0.7409 for samples BD, FS, LF, MD, CN, respectively. Furthermore, the predicted *R*-square value of 0.7432, 0.7008, 0.7402, 0.4233, and 0.5424 in BD, FS, LF, MD, and CN is also highly consistent with the adjusted *R*-square value (see Additional file 1). A model that has an *R*-square value above 0.75 is regarded as a significant and acceptable model based on the correlation between observed data and predicted data. The difference between predicted and adjusted *R*-square value was smaller than 0.2 [26].

**Fig. 5:**
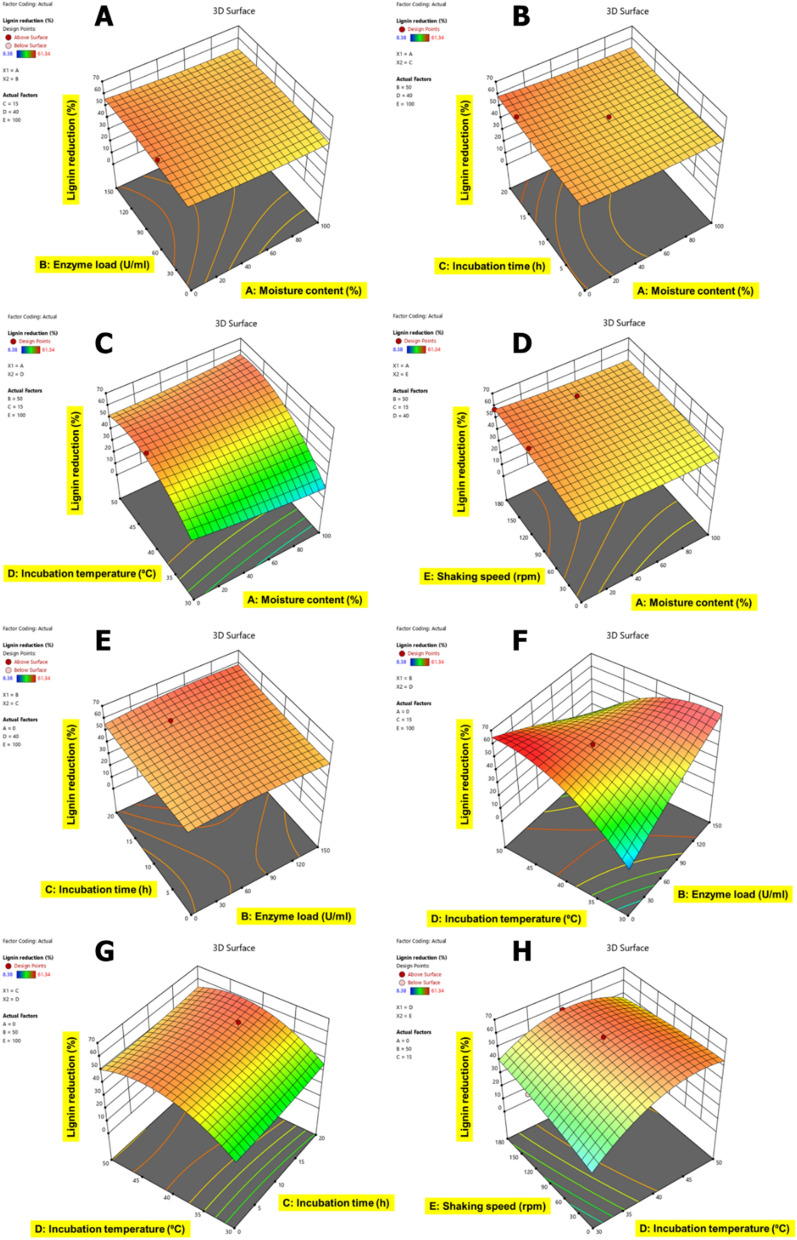
3D surface plots for the response lignin reduction (%) due to EnZolv pretreatment in blowroom droppings. **A** Effect of moisture content vs. enzyme load; **B** effect of moisture content vs. incubation time; **C** effect of moisture content versus incubation temperature; **D** effect of moisture content vs. shaking speed; **E** effect of enzyme load vs. incubation time; **F** effect of enzyme load vs. incubation temperature; **G** effect of incubation time vs. incubation temperature; **H** effect of incubation temperature vs. shaking speed. The interactive effect is represented with color ranging from blue to red (blue, green, red); blue is least significant, green is moderately significant, and red is highly significant

**Fig. 6:**
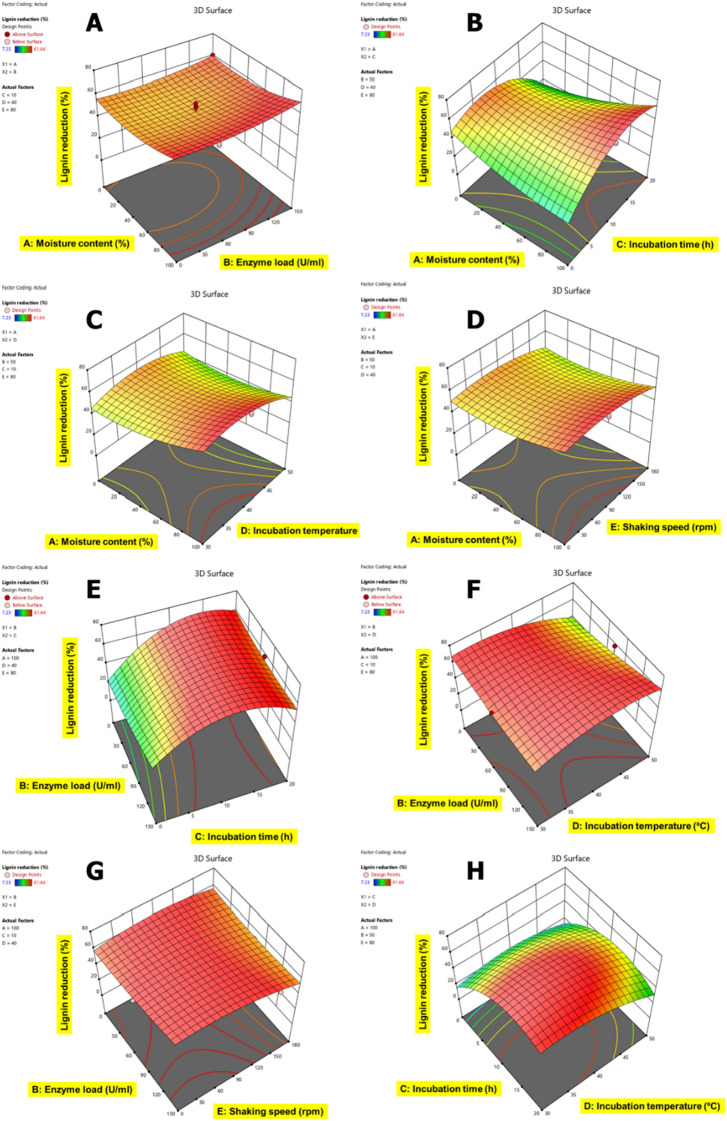
3D surface plots for the response lignin reduction (%) due to EnZolv pretreatment in flat strips. **A** Effect of moisture content vs. enzyme load; **B** effect of moisture content vs. incubation time; **C** effect of moisture content vs. incubation temperature; **D** effect of moisture content vs. shaking speed; **E** effect of enzyme load vs. incubation time; **F** effect of enzyme load vs. incubation temperature; **G** effect of enzyme load vs. shaking speed; **H** effect of incubation time vs. incubation temperature. The interactive effect is represented with color ranging from blue to red (blue, green, red); blue is least significant, green is moderately significant, and red is highly significant

**Fig. 7:**
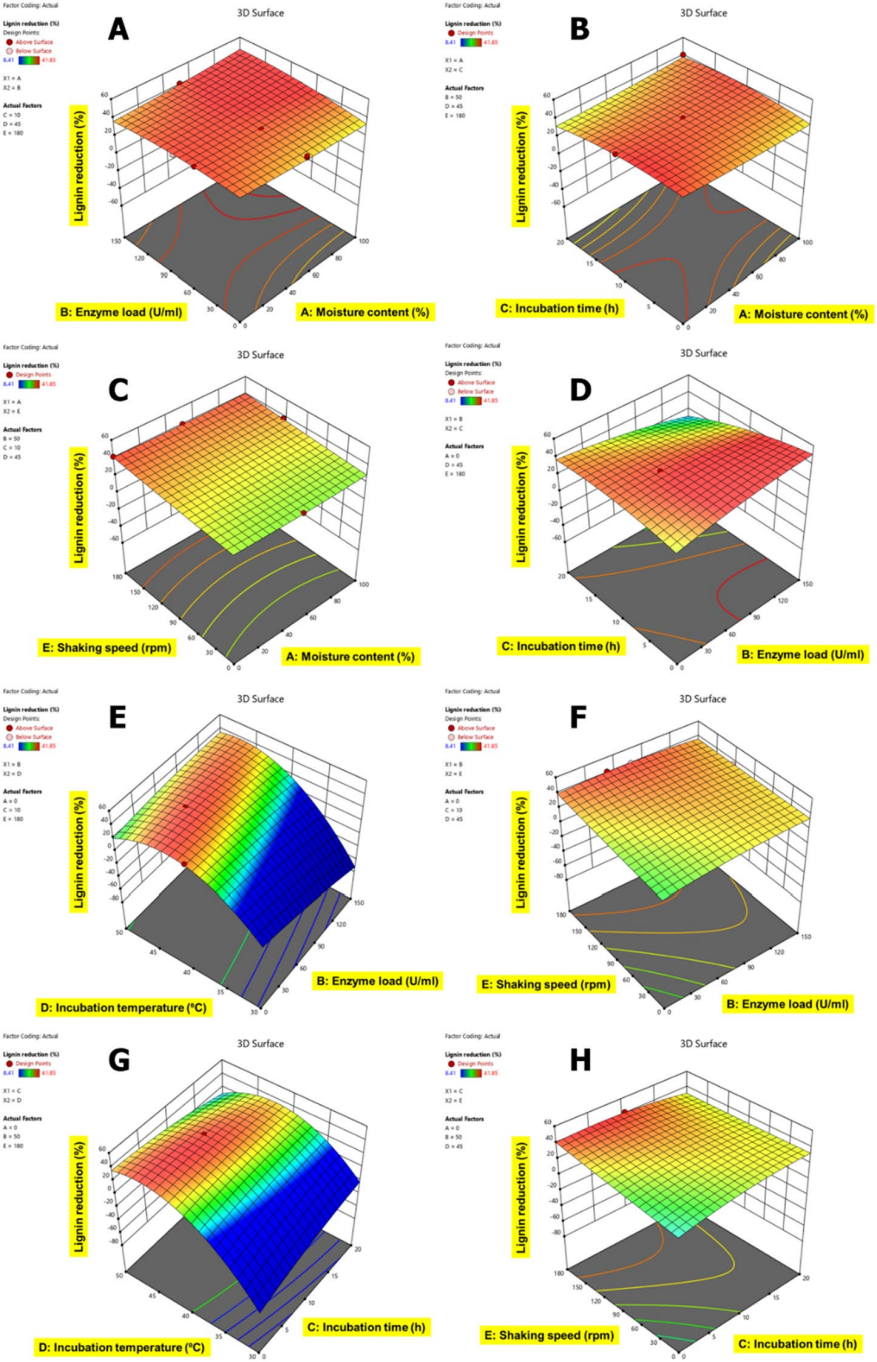
3D surface plots for the response lignin reduction (%) due to EnZolv pretreatment in lickerin fly. **A** Effect of moisture content vs. enzyme load; **B** effect of moisture content vs. incubation time; **C** effect of moisture content vs. shaking speed; **D** effect of enzyme load vs. incubation time; **E** effect of enzyme load vs. incubation temperature; **F** effect of enzyme load vs. shaking speed; **G** effect of incubation time vs. incubation temperature; **H** effect of incubation time vs. shaking speed. The interactive effect is represented with color ranging from blue to red (blue, green, red); blue is least significant, green is moderately significant, and red is highly significant

**Fig. 8:**
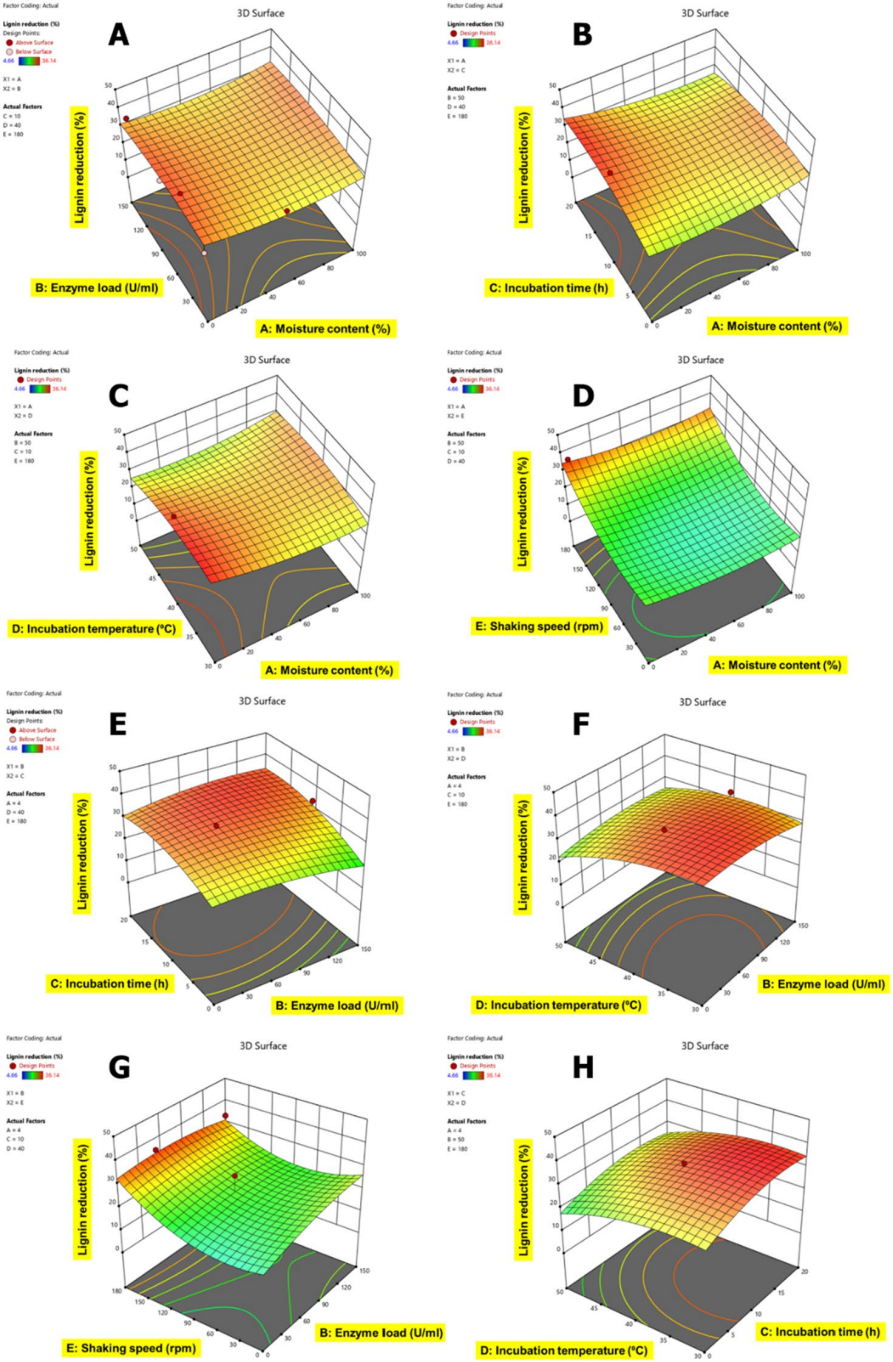
3D surface plots for the response lignin reduction (%) due to EnZolv pretreatment in microdust. **A** Effect of moisture content vs. enzyme load; **B** effect of moisture content vs. incubation time; **C** effect of moisture content vs. incubation temperature; **D** effect of moisture content vs. shaking speed; **E** effect of enzyme load vs. incubation time; **F** effect of enzyme load vs. incubation temperature; **G** effect of enzyme load vs. shaking speed; **H** effect of incubation time vs. incubation temperature. The interactive effect is represented with color ranging from blue to red (blue, green, red); blue is least significant, green is moderately significant, and red is highly significant

**Fig. 9:**
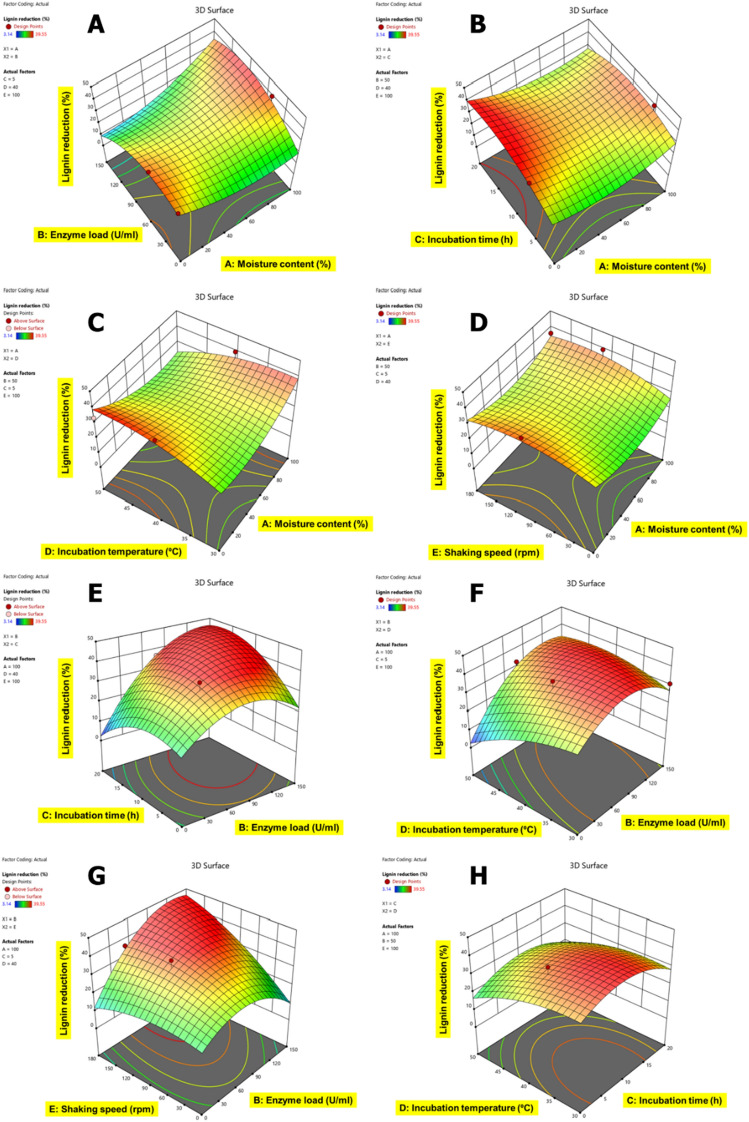
3D surface plots for the response lignin reduction (%) due to EnZolv pretreatment in comber noils. **A** Effect of moisture content vs. enzyme load; **B** effect of moisture content vs. incubation time; **C** effect of moisture content vs. incubation temperature; **D** effect of moisture content vs. shaking speed; **E** effect of enzyme load vs. incubation time; **F** effect of enzyme load vs. incubation temperature; **G** effect of enzyme load vs. shaking speed; **H** effect of incubation time vs. incubation temperature. The interactive effect is represented with color ranging from blue to red (blue, green, red); blue is least significant, green is moderately significant, and red is highly significant

The correlation of variable factors with each other for lignin content reduction in CSMW is shown in Figs. 5, 6, 7, 8 and 9. The interactive effects of model variables presented a quadratic model for all the CSMW. The best suited process parameters optimized using RSM for EnZolv pretreatment in CSMW are provided. Three-dimensional response surface plots showed the relationship of five variables at their best values. The plots had a distinct peak within the design boundary, meaning that the highest lignin removal was achievable in that range. The statistically optimized parameters in BD showed 59.16% lignin reduction at 0% moisture content, enzyme load of 75 U g^−1^, 20 h incubation, 40 °C incubation temperature, and 100 rpm shaking speed. With 100% moisture content, 50 U g^−1^ enzyme load, 10 h incubation time, incubation temperature of 40 °C, and a shaking speed of 80 rpm, the percentage of lignin reduction in FS from BBD revealed lignin reduction of 62.88%. The actual percentage of lignin reduction was determined to be 61.64% after an experiment was conducted to verify the predicted optimum circumstances. At 70.44% moisture content, 145.2 U g^−1^ enzyme load, 3.96 h incubation time, incubation temperature of 45.14 °C, and a shaking speed of 155.85 rpm, the percentage of lignin reduction in LF was 48.26%; whereas the actual percentage of lignin reduction was determined to be 41.85% after an experiment was conducted to verify the predicted optimum circumstances. At 0% moisture content, enzyme load of 75 U g^−1^ of biomass, 10 h of incubation time, incubation temperature of 40 °C, and a shaking speed of 180 rpm, the percentage of lignin reduction using the Box–Behnken design is 34.64% for MD. With a moisture content of 100%, enzyme load of 100 U g^−1^ of biomass, 10 h of incubation time, incubation temperature of 40 °C, and a shaking speed of 100 rpm, the percentage of lignin reduction was recorded to be 45.99% by optimization employing the Box–Behnken design for CN. After being verified by experimenting with the predicted ideal conditions, the experimental lignin reduction (%) was discovered to be 35.86%.

## Discussion

Lignin is a complex aromatic polymer that binds to cellulose and hemicellulose in plant cell walls, making them resistant to enzymatic hydrolysis and inhibiting subsequent fermentation [27, 28]. Pretreatment of cotton waste biomass to reduce lignin is a crucial step in the biochemical production. Several methods have been reported for lignin reduction in cotton waste biomass, such as organic acid pretreatment, ethanol-assisted hot water pretreatment, and biological pretreatment [27–30]. However, the EnZolv process parameters were already optimized for woody biomass from *Melia dubia*, and the lignin reduction was insignificant when aiming for an industrial scale [12]. In the present study, conventional EnZolv pretreatment of five different CSMW showed a lignin reduction in the range of 35–61%. The results are in agreement with lignin reduction of 45.36% and 35.02% using EnZolv pretreatment of cotton stalk (CS) and ginning mill waste (GMW), respectively [31]. A concurrent study using CSMW for pretreatment with *Pleurotus florida* exhibited lignin degradation of 60% (w/w) [32]. Contrarily, wheat straw biomass when pretreated with phosphoric acid and hydrogen peroxide exhibited a lower lignin reduction of 4.5% which was comparatively lower than EnZolv pretreated CSMW [33]. Organic acid pretreatment uses acids such as maleic acid, oxalic acid, and citric acid to solubilize hemicellulose and lignin from cotton waste biomass [29]. This method reports high lignin removal (up to 90%) and high sugar recovery (up to 80%) at optimal conditions [30]. Additionally, a blend of waste cardboard from packaging and surgical waste when pretreated with 15% v/v ammonia removed 70% of the lignin which is higher than EnZolv pretreatment. In the ammonia pretreatment process, the cleavage of the ether bond results in the separation of lignin from the polysaccharide matrix and the breakdown of the sugar polymers into monomer and lignin fragments [34]. Ethanol-assisted hot water pretreatment uses a mixture of ethanol and water to extract lignin from cotton waste biomass at high temperatures and pressures [28]. This method can produce low molecular weight lignin (about 900 Da) with a high yield (15.65%) and a typical grass lignin structure (guaiacyl, syringyl, and p-hydroxyphenyl units). The extracted lignin can also be used as an adsorbent for dye removal from wastewater [28]. Likewise, the use of EnZolv pretreatment of agro-residues aids both in delignification as well as high-value chemical generation [12]. Biological pretreatment uses microorganisms such as fungi and bacteria to degrade lignin from cotton waste biomass by producing enzymes such as laccase, peroxidase, and cellulase. This method can reduce the lignin content (up to 50%) and increase the cellulose content (up to 70%) of cotton waste biomass after several weeks of incubation [27, 29]. However, this method is slower and less efficient than EnZolv since it uses a combined strategy [27]. The advantages of EnZolv pretreatment over other methods include the generation of high-value lignin-derived phenolics, eco-friendly when released in the environment, inhibitors such as furans, and hydroxy methyl furfural (HMF) are not produced thereby increasing the efficiency of saccharification [12]. The comparison of lignin reduction due to different pretreatment methods followed for various agro-residues is described in Table 1.

**Table 1:** Lignin removal due to different pretreatment methods followed for agro-residues

Biomass	Pretreatment method	Lignin removal (%)	References
Bamboo culms	*Punctualaria* sp. TUFC20056	50	[50]
Corn stover	Fungal consortium	43.8	[51]
Corn stalk	*Irpex lacteus*	37.6	[52]
Cotton spinning mill waste	*Pleurotus florida*	60	[32]
Wheat straw	Phosphoric acid + H_2_O_2_	4.5	[33]
Sugarcane bagasse	Acetone–phenoxyethanol–water	98.1	[37]
Sugarcane bagasse	*Pycnoporus cinnabarinus*	31	[38]
Coir yarn	Alkali pretreatment	50	[48]
Peanut shell	Mild acid pretreatment (0.5%)	47.4	[45]
Corn cob	Hydrodynamic cavitation + Laccase	90	[47]
Blowroom droppings	EnZolv pretreatment	61.34	This study
Flat strips	EnZolv pretreatment	61.64	This study
Lickerin fly	EnZolv pretreatment	41.85	This study
Microdust	EnZolv pretreatment	35.34	This study
Comber noils	EnZolv pretreatment	35.86	This study

Moisture content of the biomass during pretreatment plays a critical role by making the biomass more susceptible during steam treatment thereby making the enzyme more accessible to lignin hydrolysis [35]. CSMW reported maximum lignin reduction due to moisture content optimization in EnZolv pretreatment of 11.81%, and 80.70% at 0% moisture content for BD, and LF, 60.30% at 4% moisture content for MD, and 65.22%, and 76.72% at 100% moisture content for FS, and CN, respectively. A similar study for optimizing moisture content in EnZolv pretreatment of CS and GMW revealed maximum delignification of 69.3% and 71.52% at 0% and 100% moisture content, respectively [31]. The results gained are in agreement with earlier research done by Iniyakumar and his colleagues [12]. Optimization of enzyme load in EnZolv pretreatment of CSMW showed lignin reduction in the range of 21.27%–32.40% which was in agreement with previous study using CS and GMW as biomass [31]. A buffer-free solvent medium limits the activity of enzyme in changing pH environments, and limits solubility of proteins in solvents. Moreover, solvents facilitate electron transfer and movement between the substrate in its oxidized and reduced forms and between the substrate and enzyme. This makes the fungal laccases (LccH) act on the substrate for a longer time by making the enzyme stable [12].

Pretreatment temperature and incubation time are significant determinants in determining the combined severity factor (CSF) of acid-catalyzed steam explosion from biomass of *Miscanthus* × *giganteus*, according to Auxenfans and his coworkers [36]. Incubation time optimization in CSMW due to EnZolv showed maximum lignin reduction at 5 h (CN), 10 h (FS, LF, and MD), 15 h (BD). This was evidenced by the previous findings which showed EnZolv pretreatment with maximum lignin removal at 5 h incubation time for both CS and GMW [31]. Incubation temperature of 40 °C (LF), and 45 °C (BD, FS, MD, CN) showed maximum lignin reduction due to EnZolv pretreatment which was in accordance with CS and GMW biomass that exhibited maximum removal at 50 °C and 45 °C, respectively [31]. Previous investigations using EnZolv pretreatment showed maximum lignin reduction at 150 rpm and 80 rpm for CS and GMW biomass, respectively [31]. Under ideal circumstances, pretreatment with acetone–phenoxyethanol–water resulted in the removal of 98.1% of the lignin and increased cellulose digestibility to 74.5% as opposed to raw sugarcane bagasse's (SCB) low digestibility of 9.3% from sugarcane bagasse with 0.17 M H_2_SO_4_ at 125 °C for 120 min, L/S of 15 [37]. Similar studies showed that lignin was reduced by 31% after biologically pretreating sugarcane bagasse with 50 U g^−1^ of biomass from the isolate *Pycnoporus cinnabarinus* at 170 rpm and 50 °C which was in accordance with the above finding [38]. The results of EnZolv pretreatment method in optimized incubation time and incubation temperature ensures enzyme stability and avoids expensive and severe operating conditions. Therefore, EnZolv can achieve efficient delignification and enable the recovery of valuable aromatic compounds from lignin.

It has been shown that hemicellulose may be successfully removed from a variety of feedstocks, such as switch grass, wheat straw, and maize stover, by dilute acid pretreatment [39]. Corncobs subjected to oxalic acid pretreatment decreased the hemicellulose concentration of the recovered solids from 27.86% before pretreatment to just 6.76% after pretreatment [40]. Similar studies show that pretreating rice straw with steam explosion lowers its hemicellulose content from 57% to 46.5% from the starting value of 57% [41]. Ammonia fiber expansion (AFEX) pretreatment can remove up to 40% of the hemicellulose found in biomass without using harsh chemicals or high heat [42]. Hemicellulose removal increased from 58.38% to 82.05% when pretreated with acetic acid and levulinic acid, respectively [37]. Similar studies indicate that steam explosion pretreatment of rice straw improves its cellulose content from 32.4% to as high as 54.5% [41]. Adding sodium bisulfite and 2-naphthol-7-sulfonate simultaneously to the acid pretreatment boosted the cellulose hydrolysis yield in poplar sawdust from 47.9% to 90.6% [43]. A similar study evaluated the possibility of making bioethanol from 10 distinct cotton spinning by-products. It has been demonstrated that the CSMW had a high cellulose content (55%–86% w/w). The paramount results in terms of cellulose recovery and crystallinity reduction for the pretreatment of cotton spinning wastes were achieved with 12% NaOH, 5 °C, and 3 h, which were 98% and 88%, respectively [32].

Lignin reduction is a key step in the pretreatment of biomass for bioethanol production, as it improves the accessibility and digestibility of cellulose and hemicellulose by enzymes and microorganisms [44, 45]. Various process parameters, such as time, temperature, acid or alkali concentration, mass:liquor ratio, particle size, and type of catalyst, can affect the efficiency and yield of lignin reduction from biomass pretreatment [44–48]. Different optimization methods, such as response surface methodology (RSM), technique for order preference by similarity to ideal solution (TOPSIS), and multi-objective optimization (MOO), have been applied to find the optimal values of process parameters that can maximize lignin removal and minimize negative effects on biomass properties [45, 47, 48]. Statistical optimization in the present study also simulated process parameters and the lignin reduction was exhibited as 59.16%, 62.88%, 48.26%, 34.64%, and 45.99% for the biomass BD, FS, LF, LF, and CN, respectively. Previous investigations employing RSM-based optimization of process parameters in EnZolv pretreatment showed an optimized lignin reduction of 78.68% and 70.53% in CS and GMW, respectively [31]. Likewise, the reaction parameters examined for pretreatment in empty palm fruit bunch using ethanol organosolv pretreatment were sulphuric acid concentration (0.5–2.0%), reaction temperature (160–200 °C), and residence time (45–90 min). The experimental data (96.0%) for glucose and lignin recovery were in excellent agreement with the central composite design prediction (100%), and the optimal values of the variables were as follows: sulphuric acid 2.0% w/w, 160 °C, and 78 min [49]. Mild acid pretreatment (0.5%) of peanut shell biomass at ideal conditions of mass: liquor ratio of 1:10, incubation for 1 h at 140 °C. removed 47.4% lignin, 3.2% hemicellulose, and recovered 25.3% cellulose [45]. With hydrodynamic cavitation and enzymatic pretreatment of corncob biomass under optimal values of biomass loading of 5%, enzyme loading of 6.5 U g^−1^ of biomass, at the incubation time of 60 min resulted in lignin removal of 90%, hemicellulose removal of 85%, and cellulose retention of 95% [47]. Alkali pretreatment of coir yarn with ideal conditions of mass:liquor ratio of 1:20, incubation for 1 h at 100 °C reduced 50% of the lignin [48]. Though various pretreatment methods have been developed, EnZolv is a standalone pretreatment process that supports environmental sustainability by providing renewable green chemicals from agro-residues.

## Conclusion

EnZolv pretreatment of CSMW under optimized conditions using RSM disclosed a lignin reduction of 59.16%, 62.88%, 48.26%, 36.64%, and 45.99% in BD, FS, LF, MD, and CN, respectively. EnZolv has proved to be an eco-friendly, cost-effective approach to paving a greener path for biomass-derived chemicals, biofuels, and oligosaccharides extraction. Moreover, EnZolv has accomplished the circular economy concept and biomass valorization thereby opening an endless market that encourages the bioproducts produced through agro-residues. With an endless flow of agro-residues, this alternative strategy can be employed for scaling up the process thereby proving a sustainable transition for biomass-derived products from agro-residues.

### Supplementary Information

**Additional file 1. Table S1**: ANOVA for the quadratic model of EnZolv pretreatment conditions in blowroom-dropping biomass. **Table S2**: ANOVA for the quadratic model of EnZolv pretreatment conditions in flat strips biomass. **Table S3**: ANOVA for the quadratic model of EnZolv pretreatment conditions in lickerin fly biomass. **Table S4**: ANOVA for the quadratic model of EnZolv pretreatment conditions in microdust biomass. **Table S5**: ANOVA for the quadratic model of EnZolv pretreatment conditions in comber noils biomass.

## Data Availability

The primary data that support the findings of this study are not openly available and can be obtained from the corresponding author upon reasonable request.

## Abbreviations

CSMW: Cotton spinning mill waste
BD: Blowroom droppings
FS: Flat strips
LF: Lickerin fly
MD: Microdust
CN: Comber noils
CS: Cotton stalk
GMW: Ginning mill waste
RSM: Response surface methodology
