# Supplementary material for: EnZolv delignification of cotton spinning mill waste and optimization of process parameters using response surface methodology (RSM)
Source: Biotechnol Biofuels Bioprod. 2024 Mar 7;17:37. doi: 10.1186/s13068-024-02473-w (PMC10918963; doi:10.1186/s13068-024-02473-w)
Supplement: Supplementary file 1 — Additional file 1. Table S1: ANOVA for the quadratic model of EnZolv pretreatment conditions in blowroom-dropping biomass. Table S2: ANOVA for the quadratic model of EnZolv pretreatment conditions in flat strips biomass. Table S3: ANOVA for the quadratic model of EnZolv pretreatment conditions in lickerin fly biomass. Table S4: ANOVA for the quadratic model of EnZolv pretreatment conditions in microdust biomass. Table S5: ANOVA for the quadratic model of EnZolv pretreatment conditions in comber noils biomass. [file 13068_2024_2473_MOESM1_ESM.docx]

| **Source** | **Sum of Squares** | **df** | **Mean Square** | **F-value** | ***p*-value** |  |
| --- | --- | --- | --- | --- | --- | --- |
| **Model** | 4778.62 | 20 | 238.93 | 19.21 | < 0.0001 | Significant |
| A-Moisture content | 51.71 | 1 | 51.71 | 4.16 | 0.0522 |  |
| B-Enzyme load | 5.86 | 1 | 5.86 | 0.4715 | 0.4986 |  |
| C-Incubation time | 32.94 | 1 | 32.94 | 2.65 | 0.1162 |  |
| D-Incubation temperature | 430.92 | 1 | 430.92 | 34.64 | < 0.0001 |  |
| E-Shaking speed | 79.31 | 1 | 79.31 | 6.38 | 0.0183 |  |
| AB | 0.1643 | 1 | 0.1643 | 0.0132 | 0.9094 |  |
| AC | 0.7649 | 1 | 0.7649 | 0.0615 | 0.8062 |  |
| AD | 58.03 | 1 | 58.03 | 4.67 | 0.0406 |  |
| AE | 0.0676 | 1 | 0.0676 | 0.0054 | 0.9418 |  |
| BC | 4.53 | 1 | 4.53 | 0.3643 | 0.5516 |  |
| BD | 1643.09 | 1 | 1643.09 | 132.10 | < 0.0001 |  |
| BE | 0.0027 | 1 | 0.0027 | 0.0002 | 0.9883 |  |
| CD | 0.0198 | 1 | 0.0198 | 0.0016 | 0.9685 |  |
| CE | 5.19 | 1 | 5.19 | 0.4175 | 0.5241 |  |
| DE | 178.04 | 1 | 178.04 | 14.31 | 0.0009 |  |
| A² | 9.09 | 1 | 9.09 | 0.7306 | 0.4008 |  |
| B² | 30.67 | 1 | 30.67 | 2.47 | 0.1290 |  |
| C² | 12.69 | 1 | 12.69 | 1.02 | 0.3222 |  |
| D² | 1283.47 | 1 | 1283.47 | 103.19 | < 0.0001 |  |
| E² | 18.81 | 1 | 18.81 | 1.51 | 0.2303 |  |
| **Residual** | 310.96 | 25 | 12.44 |  |  |  |
| Lack of Fit | 310.71 | 24 | 12.95 | 51.36 | 0.1098 | Not significant |
| Pure Error | 0.2521 | 1 | 0.2521 |  |  |  |
| **Cor Total** | 5089.58 | 45 |  |  |  |  |
| **S.D** | 3.53 |  | **R^2^** |  | 0.9389 |  |
| **Mean** | 48.69 |  | **Adj. R^2^** |  | 0.8900 |  |
| **Coefficient of Variance (CV)** | 7.24 |  | **Pred. R^2^** |  | 0.7432 |  |

**Table S1:** ANOVA for the quadratic model of EnZolv pretreatment conditions in blowroom droppings biomass

| **Source** | **Sum of Squares** | **df** | **Mean Square** | **F-value** | ***p*-value** |  |
| --- | --- | --- | --- | --- | --- | --- |
| **Model** | 14558.55 | 20 | 727.93 | 14.65 | < 0.0001 | Significant |
| A-Moisture content | 235.47 | 1 | 235.47 | 4.74 | 0.0391 |  |
| B-Enzyme load | 74.66 | 1 | 74.66 | 1.50 | 0.2317 |  |
| C-Incubation time | 75.67 | 1 | 75.67 | 1.52 | 0.2287 |  |
| D-Incubation temperature | 9.30 | 1 | 9.30 | 0.1871 | 0.6691 |  |
| E-Shaking speed | 291.24 | 1 | 291.24 | 5.86 | 0.0231 |  |
| AB | 1.13 | 1 | 1.13 | 0.0227 | 0.8813 |  |
| AC | 283.30 | 1 | 283.30 | 5.70 | 0.0248 |  |
| AD | 32.94 | 1 | 32.94 | 0.6628 | 0.4233 |  |
| AE | 0.0613 | 1 | 0.0613 | 0.0012 | 0.9723 |  |
| BC | 147.60 | 1 | 147.60 | 2.97 | 0.0972 |  |
| BD | 324.87 | 1 | 324.87 | 6.54 | 0.0170 |  |
| BE | 70.92 | 1 | 70.92 | 1.43 | 0.2434 |  |
| CD | 288.37 | 1 | 288.37 | 5.80 | 0.0237 |  |
| CE | 60.32 | 1 | 60.32 | 1.21 | 0.2811 |  |
| DE | 61.76 | 1 | 61.76 | 1.24 | 0.2755 |  |
| A² | 280.82 | 1 | 280.82 | 5.65 | 0.0254 |  |
| B² | 79.75 | 1 | 79.75 | 1.60 | 0.2169 |  |
| C² | 2637.05 | 1 | 2637.05 | 53.07 | < 0.0001 |  |
| D² | 634.47 | 1 | 634.47 | 12.77 | 0.0015 |  |
| E² | 303.54 | 1 | 303.54 | 6.11 | 0.0206 |  |
| **Residual** | 1242.30 | 25 | 49.69 |  |  |  |
| Lack of Fit | 1219.51 | 21 | 58.07 | 10.20 | 0.0181 | Not significant |
| Pure Error | 22.78 | 4 | 5.70 |  |  |  |
| **Cor Total** | 15800.85 | 45 |  |  |  |  |
| **S.D** | 7.05 |  | **R^2^** |  | 0.9214 |  |
| **Mean** | 40.37 |  | **Adj. R^2^** |  | 0.8585 |  |
| **Coefficient of Variance (CV)** | 17.46 |  | **Pred. R^2^** |  | 0.7008 |  |

**Table S2:** ANOVA for the quadratic model of EnZolv pretreatment conditions in flat strips biomass

| **Source** | **Sum of Squares** | **df** | **Mean Square** | **F-value** | ***p*-value** |  |
| --- | --- | --- | --- | --- | --- | --- |
| **Model** | 3136.37 | 20 | 156.82 | 16.10 | < 0.0001 | Significant |
| A-Moisture content | 7.99 | 1 | 7.99 | 0.8204 | 0.3737 |  |
| B-Enzyme load | 36.56 | 1 | 36.56 | 3.75 | 0.0641 |  |
| C-Incubation time | 276.25 | 1 | 276.25 | 28.35 | < 0.0001 |  |
| D-Incubation temperature | 502.22 | 1 | 502.22 | 51.55 | < 0.0001 |  |
| E-Shaking speed | 22.32 | 1 | 22.32 | 2.29 | 0.1427 |  |
| AB | 33.01 | 1 | 33.01 | 3.39 | 0.0776 |  |
| AC | 35.04 | 1 | 35.04 | 3.60 | 0.0695 |  |
| AD | 0.1806 | 1 | 0.1806 | 0.0185 | 0.8928 |  |
| AE | 0.0768 | 1 | 0.0768 | 0.0079 | 0.9299 |  |
| BC | 243.27 | 1 | 243.27 | 24.97 | < 0.0001 |  |
| BD | 358.67 | 1 | 358.67 | 36.81 | < 0.0001 |  |
| BE | 52.73 | 1 | 52.73 | 5.41 | 0.0284 |  |
| CD | 297.87 | 1 | 297.87 | 30.57 | < 0.0001 |  |
| CE | 106.86 | 1 | 106.86 | 10.97 | 0.0028 |  |
| DE | 21.06 | 1 | 21.06 | 2.16 | 0.1540 |  |
| A² | 6.88 | 1 | 6.88 | 0.7064 | 0.4086 |  |
| B² | 74.85 | 1 | 74.85 | 7.68 | 0.0104 |  |
| C² | 148.53 | 1 | 148.53 | 15.24 | 0.0006 |  |
| D² | 644.71 | 1 | 644.71 | 66.17 | < 0.0001 |  |
| E² | 0.4582 | 1 | 0.4582 | 0.0470 | 0.8301 |  |
| **Residual** | 243.58 | 25 | 9.74 |  |  |  |
| Lack of Fit | 242.51 | 22 | 11.02 | 30.96 | 0.0080 | Not significant |
| Pure Error | 1.07 | 3 | 0.3560 |  |  |  |
| **Cor Total** | 3379.94 | 45 |  |  |  |  |
| **S.D** | 3.12 |  | **R^2^** |  | 0.9279 |  |
| **Mean** | 30.75 |  | **Adj. R^2^** |  | 0.8703 |  |
| **Coefficient of Variance (CV)** | 10.15 |  | **Pred. R^2^** |  | 0.7402 |  |

**Table S3:** ANOVA for the quadratic model of EnZolv pretreatment conditions in lickerin fly biomass

| **Source** | **Sum of Squares** | **df** | **Mean Square** | **F-value** | ***p*-value** |  |
| --- | --- | --- | --- | --- | --- | --- |
| **Model** | 3009.62 | 20 | 150.48 | 4.56 | 0.0002 | Significant |
| A-Moisture content | 7.43 | 1 | 7.43 | 0.2251 | 0.6393 |  |
| B-Enzyme load | 84.09 | 1 | 84.09 | 2.55 | 0.1231 |  |
| C-Incubation time | 10.79 | 1 | 10.79 | 0.3267 | 0.5727 |  |
| D-Incubation temperature | 4.27 | 1 | 4.27 | 0.1293 | 0.7222 |  |
| E-Shaking speed | 424.94 | 1 | 424.94 | 12.87 | 0.0014 |  |
| AB | 6.57 | 1 | 6.57 | 0.1990 | 0.6593 |  |
| AC | 20.24 | 1 | 20.24 | 0.6130 | 0.4410 |  |
| AD | 45.07 | 1 | 45.07 | 1.36 | 0.2537 |  |
| AE | 0.1263 | 1 | 0.1263 | 0.0038 | 0.9512 |  |
| BC | 13.54 | 1 | 13.54 | 0.4100 | 0.5278 |  |
| BD | 15.25 | 1 | 15.25 | 0.4619 | 0.5030 |  |
| BE | 33.88 | 1 | 33.88 | 1.03 | 0.3208 |  |
| CD | 0.0022 | 1 | 0.0022 | 0.0001 | 0.9935 |  |
| CE | 0.1604 | 1 | 0.1604 | 0.0049 | 0.9450 |  |
| DE | 20.68 | 1 | 20.68 | 0.6262 | 0.4362 |  |
| A² | 63.62 | 1 | 63.62 | 1.93 | 0.1774 |  |
| B² | 47.07 | 1 | 47.07 | 1.43 | 0.2437 |  |
| C² | 85.08 | 1 | 85.08 | 2.58 | 0.1210 |  |
| D² | 111.91 | 1 | 111.91 | 3.39 | 0.0775 |  |
| E² | 388.63 | 1 | 388.63 | 11.77 | 0.0021 |  |
| **Residual** | 825.55 | 25 | 33.02 |  |  |  |
| Lack of Fit | 705.00 | 22 | 32.05 | 0.7975 | 0.6856 | Not significant |
| Pure Error | 120.55 | 3 | 40.18 |  |  |  |
| **Cor Total** | 3835.18 | 45 |  |  |  |  |
| **S.D** | 5.75 |  | **R^2^** |  | 0.7847 |  |
| **Mean** | 21.26 |  | **Adj. R^2^** |  | 0.6125 |  |
| **Coefficient of Variance (CV)** | 27.04 |  | **Pred. R^2^** |  | 0.4233 |  |

**Table S4:** ANOVA for the quadratic model of EnZolv pretreatment conditions in microdust biomass

| **Source** | **Sum of Squares** | **df** | **Mean Square** | **F-value** | ***p*-value** |  |
| --- | --- | --- | --- | --- | --- | --- |
| **Model** | 6639.61 | 20 | 331.98 | 7.43 | < 0.0001 | Significant |
| A-Moisture content | 10.54 | 1 | 10.54 | 0.2359 | 0.6314 |  |
| B-Enzyme load | 15.98 | 1 | 15.98 | 0.3578 | 0.5551 |  |
| C-Incubation time | 362.05 | 1 | 362.05 | 8.11 | 0.0087 |  |
| D-Incubation temperature | 93.65 | 1 | 93.65 | 2.10 | 0.1601 |  |
| E-Shaking speed | 794.83 | 1 | 794.83 | 17.79 | 0.0003 |  |
| AB | 316.86 | 1 | 316.86 | 7.09 | 0.0133 |  |
| AC | 26.55 | 1 | 26.55 | 0.5945 | 0.4479 |  |
| AD | 210.95 | 1 | 210.95 | 4.72 | 0.0394 |  |
| AE | 7.10 | 1 | 7.10 | 0.1589 | 0.6935 |  |
| BC | 295.50 | 1 | 295.50 | 6.62 | 0.0164 |  |
| BD | 119.58 | 1 | 119.58 | 2.68 | 0.1143 |  |
| BE | 257.39 | 1 | 257.39 | 5.76 | 0.0241 |  |
| CD | 8.16 | 1 | 8.16 | 0.1827 | 0.6727 |  |
| CE | 46.96 | 1 | 46.96 | 1.05 | 0.3150 |  |
| DE | 89.28 | 1 | 89.28 | 2.00 | 0.1698 |  |
| A² | 330.16 | 1 | 330.16 | 7.39 | 0.0117 |  |
| B² | 1148.78 | 1 | 1148.78 | 25.72 | < 0.0001 |  |
| C² | 506.51 | 1 | 506.51 | 11.34 | 0.0025 |  |
| D² | 76.53 | 1 | 76.53 | 1.71 | 0.2024 |  |
| E² | 152.15 | 1 | 152.15 | 3.41 | 0.0768 |  |
| **Residual** | 1116.68 | 25 | 44.67 |  |  |  |
| Lack of Fit | 1086.26 | 24 | 45.26 | 1.49 | 0.5796 | Not significant |
| Pure Error | 30.42 | 1 | 30.42 |  |  |  |
| **Cor Total** | 7756.29 | 45 |  |  |  |  |
| **S.D** | 6.68 |  | **R^2^** |  | 0.8560 |  |
| **Mean** | 24.20 |  | **Adj. R^2^** |  | 0.7409 |  |
| **Coefficient of Variance (CV)** | 27.62 |  | **Pred. R^2^** |  | 0.5224 |  |

**Table S5:** ANOVA for the quadratic model of EnZolv pretreatment conditions in comber noils biomass
